# Current Concepts in the Prevention of Perioperative Myocardial Injury

**DOI:** 10.31480/2330-4871/127

**Published:** 2020-08-04

**Authors:** Christian Bohringer, Duc Le, Hong Liu

**Affiliations:** Department of Anesthesiology and Pain Medicine, University of California Davis Health, Sacramento, California, USA

**Keywords:** Perioperative myocardial injury, sympathetic activity, tachycardia

## Abstract

Perioperative myocardial injury is frequently caused by tachycardia from excessive sympathetic nervous system activity resulting from the surgical stimulation (type 2) rather than by rupture of atherosclerotic plaques with superimposed thrombosis (type 1). The elevated sympathetic nervous system activity results in tachycardia that induces demand ischemia within the myocardium and damages the heart muscle. A rise in troponin has been shown to be a reliable predictor of adverse cardiovascular events when measured in a population at risk. This holds true even when the troponin rise is isolated and other markers for myocardial damage like prolonged ischemic type chest pain, new electrocardiogram changes or evidence of new myocardial damage on echocardiography and other cardiac imaging studies are absent. Treatments that prevent tachycardia by successfully controlling elevated sympathetic tone, like dexmedetomidine and thoracic epidural blockade with local anesthetic reduce troponin release and have been shown to prevent myocardial damage. Intravenous lidocaine and magnesium can also prevent tachycardia. Beta blockers reduce myocardial injury, but are associated with an increase in hypotension and ischemic stroke. Any method of attenuating sympathetic nervous system activity, may however, require treatment with intravenous fluids and vasopressors to prevent hypotension. Rupture of atherosclerotic plaques with superimposed coronary thrombosis is a far less common cause of myocardial infarction in the perioperative period than elevated sympathetic tone. This explains why prophylactic statins in previously statin-naïve patients do not reduce major adverse cardiovascular event rates. Antiplatelet agents are also ineffective in reducing adverse cardiovascular events in the perioperative period. Clinicians, therefore, need to focus their attention on heart rate control and the attenuation of the stress response to surgery, rather than on atherosclerotic plaque stability and antiplatelet drugs in order to successfully prevent perioperative myocardial injury.

## Introduction

A number of different strategies have been employed to prevent myocardial injury (MI) in the perioperative period. It has been postulated as early as 2001 that perioperative myocardial infarction is primarily caused by prolonged stress-induced myocardial ischemia [1]. This theory was developed because tachycardia usually precedes ischemic electrocardiogram (ECG) changes in the perioperative period. These ischemic ECG changes typically present as ST segment depression rather than elevation, and usually are the non-Q wave infarction rather than the Q-wave type. When coronary angiography is subsequently performed for the evaluation of perioperative myocardial ischemia, coronary thrombus and ruptured plaques are usually absent [1]. This is often called type 2 myocardial injury, which is due to a mismatch between myocardial supply and demand, exacerbated by excessive tachycardia from inadequately controlled sympathetic nervous system activity. This is the main mechanism of perioperative adverse cardiac events [2–4]. Type 1 myocardial injury resulting from atherosclerotic plaque rupture and coronary thrombosis are far less common than demand ischemia in the perioperative period [5]. This explains why treatments that control sympathetic tone are more effective in preventing perioperative MI in the operating and recovery rooms. Treatment with statins, antiplatelet agents and other anticoagulants, on the other hand, is more effective for the treatment of acute myocardial ischemia in the emergency room and the coronary care unit where the excessive sympathetic nervous system activity from the stress response to surgery is absent. In some randomized prospective trials statins and antiplatelet drugs have in fact failed to prevent perioperative MI [6,7].

**Figure 1:**
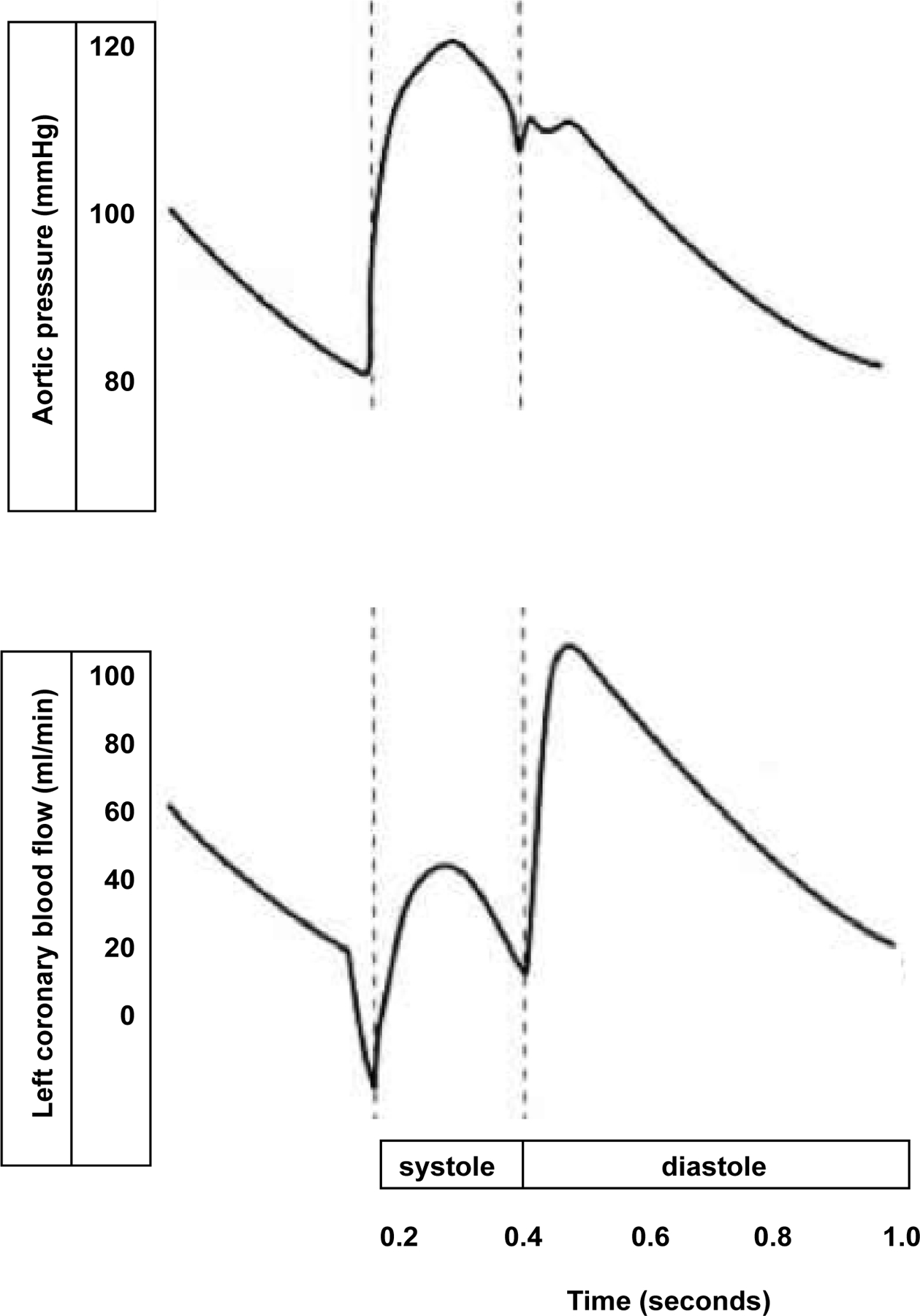
Schematic illustration that most of the blood flow through the left coronary artery takes place in diastole. The left ventricle is critically dependent on a low heart rate to provide for an adequate time in diastole when myocardial perfusion of the left ventricle takes place.

The left ventricle (LV) is the only organ in the body that provides its own blood supply. Myocardial blood flow takes place mainly in diastole when the left ventricle relaxes sufficiently for perfusion to occur. The perfusion of LV myocardium is critically dependent on heart rate. If the heart rate is too high, the time in diastole is too short and demand ischemia will occur. Aortic root pressure during diastole is the driving pressure for coronary perfusion. After heart rate has been controlled, an adequate aortic root pressure during diastole is the second most important hemodynamic parameter that needs to be preserved to prevent demand ischemia (Figure 1).

## Control of Excessive Sympathetic Nervous System Activity

### Prevention of hypotension and ischemic stroke

All treatment modalities that blunt the sympathetic nervous system response also reduce the capacity of the body to compensate for blood loss or vasodilation. Adequate circulating blood volume and normal vascular tone will need to be maintained to prevent hypotension irrespective of which type of therapy is used to reduce sympathetic nervous system tone. Adequate fluid re-suscitation and vasopressor drugs are, therefore, often required to prevent or treat hypotension after the sympathetic blockade has been established.

### Dexmedetomidine

Dexmedetomidine is a centrally acting alpha-2 agonist that acts on the presynaptic nerve terminal of the adrenergic synapse and prevents the release of noradrenaline via a negative feedback inhibition. Several trials have found it to be associated with a decreased risk of myocardial ischemia and a reduced level of troponin and other biomarkers for myocardial damage in the perioperative period [8–10]. Unlike clonidine, which is a drug useful for treating hypertension, dexmedetomidine is much more selective for the presynaptic alpha-2 receptor and is not associated with a major drop in blood pressure [11]. The significant hypotension seen with clonidine may be the reason why it has failed to prevent perioperative MI in a number of studies [12–14]. Dexmedetomidine does not impair myocardial contractility on echocardiographic evaluation [15]. It is preferable to beta-blockers for heart rate control in patients with reduced ejection fraction. Dexmedetomidine is also a powerful bronchodilator [16]. There are three mechanisms for its bronchodilator effect: antagonizing acetylcholine at the postganglionic nerve ending of the vagus nerve, producing direct relaxation of bronchial smooth muscle, and inhibiting substance P release by the C-fibers [17]. Dexmedetomidine is, therefore, preferred over beta-blockers for heart rate control in patients with asthma and chronic obstructive pulmonary disease (COPD), where beta-blocker use may cause concomitant bronchoconstriction from beta-2 adrenergic receptor blockade. Dexmedetomidine not only blocks the release of catecholamines, but also suppresses stress hormones like cortisol and growth hormone. This effectively attenuates the rise in blood glucose that is usually seen with surgery. It also blocks the release of pro-inflammatory cytokines like interleukin 6, interleukin 1B and tumor necrosis factor alpha. This leads to an improved innate and adaptive immune response [18–20]. Reduced catecholamine levels prevent the alpha adrenergic-induced prothrombotic effect on platelets and may reduce the occurrence of thrombotic events. This improved immune function may have positive effects on wound infection and cancer recurrence rates after surgery. This attenuation of the immunosuppressant effect of surgery is another advantage of dexmedetomidine over beta blockers because beta blockers merely block the catecholamine induced tachycardia without altering the hormonal stress response to surgery [21].

### Centro-neuraxial blocks

Spinal and epidural anesthesia have been shown to be associated with a 33% reduction in the myocardial infarction rate and a 50% reduction in the occurrence of deep venous thrombosis and pulmonary embolism [22]. These favorable outcomes occur because thoracic epidural anesthesia not only controls tachycardia, but also attenuates the surgery-induced stress response [23]. Erector spinae blocks are now advocated to replace thoracic epidural blockade to reduce the incidence of hypotension [24–26]. In patients at risk for myocardial ischemia, however, the sympathetic blockade provided by a thoracic epidural block is highly desirable because it reduces the risk of MI.

### Beta-blockers

Beta blockers have been shown to be associated with a favorable small decrease in non-fatal myocardial infarction, but also an unfavorable increase in non-fatal ischemic strokes as demonstrated in the POISE (Perioperative Ischemic Evaluation trial) [27]. The increased ischemic stroke rate with beta blockade was confirmed in two other studies [28,29]. This increased stroke rate could potentially have been avoided by treating patients proactively with fluids and vasopressors to prevent hypotension. It is important to realize that all treatments that block sympathetically mediated tachycardia also carry this inherent risk of hypotension and ischemic stroke. Intravenous fluids and vasopressors should be administered to patients that are hypotensive following sympathetic blockade. Beta blockers are negative inotropes and can induce bronchospasm through its beta-2 antagonism. However, beta-1 selective medications can still cause a statistically significant decrease in FEV1 depending on the dosage given, especially in susceptible patients with a high basal cholinergic tone [30]. Therefore, they are not recommended in high doses in patients with systolic heart failure or with COPD. Unlike dexmedetomidine and thoracic epidural blockade with local anesthetics, beta blockers do not have a favorable effect of attenuating the stress response to surgery. [21] They also do not control pain. In light of this available evidence, it is clear that dexmedetomidine and thoracic epidural blockade are preferred over beta blockade as a means of heart rate control in most patients. Beta blockers should only be used when other treatments to attenuate sympathetic nervous system activity have failed to achieve adequate heart rate control. They should no longer be used prophylactically in patients who are not tachycardic and hypertensive.

### Lidocaine and Magnesium

Intravenous lidocaine blocks sympathetic nervous system mediated tachycardia and hypertension [31]. It has been shown to reduce infarct size and improved survival in myocardial ischemia animal models [32,33]. It also attenuates the stress response to surgery. A study of women undergoing caesarian section under general anesthesia found reduced heart rate as well as reduced cortisol levels [34]. Intravenous magnesium also blocks sympathetic nervous system mediated tachycardia and hypertension, and reduces myocardial ischemia/reperfusion (I/R) injury and infarct size in animal models [35,36].

### Opioids

Opioids exhibit a cardioprotective effect when given in doses high enough to prevent tachycardia. Remifentanil in particular, has been shown to reduce troponin release [37,38]. Respiratory depression and ileus limit the usefulness of opioids with longer half-lives. When opiates are administered in higher doses, they frequently trigger the need for postoperative ventilation, and thus puts the patient at risk for ventilator associated complications. Peripheral mu opioid receptor antagonists (PAMORAs) can only ameliorate, but not reliably eliminate opioid induced ileus [39]. Dexmedetomidine resulted in lower coronary sinus lactate and CKMB levels during cardiac surgery than remifentanil, and the authors concluded that dexmedetomidine has a greater cardioprotective effect than remifentanil [40]. Remifentanil is also associated with the development of acute tolerance to opioids and the phenomenon of opioid induced hyperalgesia [41–43]. Analgesic drugs with longer half-lives like dexmedetomidine, lidocaine, magnesium, or hydromorphone should be administered when discontinuing the remifentanil infusion at the end of surgery to prevent rebound tachycardia and hypertension that may precipitate myocardial injury. Opioids also exacerbate the immunosuppressive effect of surgery by impairing the migration and function of white cells [44]. They have also been associated with increased morbidity and mortality due to the development of infection and cancer progression in preclinical study [45].

### Further strategies to prevent excessive sympathetic stimulation

Preoperative anxiolysis is important, and dexmedetomidine is superior to midazolam for this purpose. Dexmedetomidine is associated with a lower heart rate when compared to midazolam [46]. Maintaining a normal blood volume and body temperature are also essential to prevent unnecessary activation of the sympathetic nervous system. Minimal invasive surgery has been shown to be effective in reducing MI. Endovascular abdominal aortic aneurysm (AAA) repair, for example, has been shown to be associated with a lower rate of perioperative cardiac arrythmias, myocardial ischemia, troponin T release, cardiac events, and all-cause mortality compared with open repair [47]. Providing adequate postoperative pain relief is also of high priority when trying to prevent sympathetically mediated tachycardia (Table 1).

## Prevention of Coronary Artery Thrombosis

### Atherosclerotic plaque stabilization

The β-Hydroxy β-methylglutaryl-CoA (HMG-CoA) reductase inhibitors, also known as statins, are used in primary care medicine for their lipid lowering actions. They also have very important pleiotropic effects. They improve vascular endothelial dysfunction and have anti-inflammatory actions [48,49]. Statins also stabilize atherosclerotic plaques in arteries [50,51] and have been shown to be effective in the secondary prevention of vascular events [52]. Their efficacy in primary prevention of cardiovascular events has not been proven yet and caution should be taken for patients at low cardiovascular risk [53], however, studies have shown a reduction in adverse cardiac events in the perioperative setting [54–56]. The Vision study, which was an international prospective cohort study, also found that preoperative use of statins was associated with a lower rate of cardiovascular mortality and MINS (myocardial injury after non-cardiac surgery) [57]. A Cochrane study of 178 patients found a failure of statins to decrease the risk of mortality or myocardial infarction within 30 days after the surgery [58]. The randomized prospective LOAD (Lowering the risk of Operative complications using Atorvastatin loading Dose) trial also failed to show a reduction in major cardiovascular complications when a short-term course of atorvastatin was initiated in statin-naïve patients undergoing non-cardiac surgery [6]. Initiating statins in statin-naïve patients undergoing non-cardiac surgery needs to be evaluated with further trials [59]. This failure of atorvastatin to reduce cardio-vascular events in the LOAD trial is not really surprising, given that most perioperative MI is precipitated by sympathetically mediated tachycardia from the stress response to surgery rather than by plaque instability [1]. The current evidence suggests that perioperative statins may have a protective effect to prevent MINS, but not as effective as other methods that control tachycardia and the stress response to surgery.

### Antiplatelet therapy

The POISE-2 study, in which aspirin was administered before surgery and throughout the early postsurgical period, showed that aspirin had no significant effect on the rate of death or non-fatal myocardial infarction, but an increased the risk of major bleeding. This was also demonstrated in a previous study [7,60]. Even when aspirin is combined with clopidogrel as dual antiplatelet therapy, there is no reduction in myocardial infarction, stroke or mortality at 30 days [61]. However, the discontinuation of aspirin is associated with a significant increase in major adverse cardiac events in patients with coronary stents [62]. The continuation of antiplatelet therapy has been recommended in patients with a recent coronary stent undergoing non-cardiac surgery, except in intracranial and intraspinal procedures, as well as prostatectomy [63].

## Transfusion Threshold

A prospective study in patients at high risk for cardiac events showed no increase in adverse events with a restrictive transfusion policy of accepting a hemoglobin concentration as low as 8g/dL [64]. A meta-analysis did find increased complications when high risk patients had major surgery [65]. Clinical judgement is necessary to determine the transfusion threshold. The anesthesiologist should consider liberal transfusion strategy in high risk patients when hemodynamic instability or excessive tachycardia occurs.

### Preoperative coronary revascularization

Preoperative coronary revascularization in patients with stable ischemic heart disease has repeatedly failed to improve outcome [66,67]. Preoperative coronary revascularization should only be performed if it is indicated by the patient’s clinical state. Any plans for future non-cardiac surgery should not be taken into consideration when determining if revascularization is necessary. Percutaneous coronary interventions should be limited to patients with unstable coronary disease. If a stent has been inserted recently, there is an ongoing risk of stent thrombosis and antiplatelet therapy should be continued throughout the perioperative period [29]. Chronic statin and beta blocker therapy should be continued because their withdrawal has been associated with an increased rate of adverse events [68]. The lack of efficacy of preoperative revascularization and the success of techniques that control perioperative tachycardia by blunting the stress response to surgery have led to reduction in the number of preoperative coronary angiographies that are being performed in asymptomatic patients. Whenever a post-operative death from myocardial infarction occurs, it is important to remember that scientific data indicates that preoperative revascularization would not have prevented it. Greater attention should have been paid to control the sympathetic response to surgery, and to the monitoring and correction of hemodynamic parameters in the postoperative period.

### Early identification of myocardial injury

A perioperative rise in troponin has been shown to be of prognostic significance when measured in a population at risk for perioperative MI [69]. Elevated troponin T values strongly predicted 30-day mortality, and higher peak troponin values were associated with a shorter time to death [70]. Another study also found that a postoperative troponin rise was associated with a six-fold increase in mortality within the first year after noncardiac surgery [71]. An isolated troponin rise, without ischemic chest pain or evidence of new MI on ECG or cardiac imaging studies, is now designated as a MINS rather than an infarction. The 30-day mortality in patients with an isolated troponin rise (MINS) was comparable to that of patients with at least one other criterion for myocardial infarction, such as ischemic chest pain, new ECG changes, or imaging evidence of loss of viable myocardium [70]. There is an ongoing debate over what constitutes a perioperative myocardial infarction, because patients with an isolated troponin rise have equally bad outcomes as those who also have one of the other diagnostic criteria that qualify them for the diagnosis of myocardial infarction [72]. In view of this knowledge, an isolated troponin rise in a patient judged to be at risk for MI should be taken seriously, with initiation of appropriate hemodynamic monitoring and secondary preventive treatments. Study confirmed the increased 30-day mortality in MINS patients and found that MINS occurred in as many as 20% of vascular surgical patients. Most patients were asymptomatic and would have gone undetected without postoperative troponin measurement [73]. Unfortunately, the significance of MINS remains widely unrecognized [74].

It is now recommended for patients who are 65 years or older to have troponin measured on post-operative day 1, 2, and 3 while they are still in the hospital to avoid missing a diagnosis of MI [75]. If there is a troponin rise, then the diagnosis of MINS has been confirmed and secondary prevention measures should be implemented. Patients with MINS should be admitted to an intensive care or high dependency unit, where their heart rate and blood pressure are assessed frequently, and treatment of abnormal hemodynamic parameters can take place rapidly. A troponin assay in the post anesthesia care unit (PACU) can be used to determine whether patients should be sent to a high dependency or intensive care unit (ICU). Myocardial infarctions commonly occur on day 2 or 3 after the surgery, which may be difficult to diagnose on the standard medical-surgical ward where the patient’s heart rate and blood pressure are measured only occasionally. It is of note that in the POISE study, most of the patients that died of myocardial infarction were not admitted to an ICU. Outcomes in patients at risk from MI may be improved by providing better hemodynamic monitoring and treatment in the two to three days following the surgery when most adverse cardiac events occur. Ideally, patients should have a troponin assay and an ECG in the PACU that should be compared with the preoperative ECG. If there has been a troponin rise or a new ECG change, then the patient should be sent to an ICU or high dependency unit. Troponin levels may also be raised in conditions other than MI, and raised levels associated with these etiologies have also been associated with increased mortality [76]. It has been postulated that elevated troponin may indicate serious illness rather than MI alone [77]. Renal failure, sepsis, pulmonary embolism, burns and amyloidosis, for example, are also associated with elevated troponin [78]. Postoperative troponin levels should only be measured in patients at risk for MI, because elevated troponin levels in patients without coronary disease have not been shown to be predictive of a poor outcome. In patients undergoing living donor liver transplantation, for example, a rise in troponin was not predictive of early and 1-year mortality [79].

### Secondary prevention after MINS has been identified

The direct thrombin inhibitor dabigatran has been shown in the MANAGE trial to prevent major postoperative cardiovascular complications in MINS patients without causing a significant increase in major bleeding [80]. The dabigatran dose used was 110mg orally twice a day. A specific reversal agent for dabigatran is now available and can be used in case of bleeding complications. The reversal agent is a humanized monoclonal antibody fragment and is called idarucizumab [81]. Statins may also be of value and beta blockers should be considered for patients who are tachycardic and hypertensive.

## Summary

Perioperative myocardial injury is caused more frequently by demand ischemia than plaque instability. Anesthesia techniques that target heart rate control and reduce the stress response to surgery have been shown to be more effective in preventing perioperative MI than statins and antiplatelet drugs. Dexmedetomidine, centro-neuraxial blocks, IV lidocaine, and magnesium control heart rate and should be used in high risk patients. All methods of preventing tachycardia by attenuating sympathetic nervous system activity carry an inherent risk of hypotension. Intravenous fluids and vasopressors may be needed to prevent it. Beta blockers are associated with ischemic stroke and should only be used when the other protective therapies have failed to adequately control the heart rate. Troponin levels should be measured in high risk patients and in patients with persistent tachycardia to identify which patients will require more comprehensive postoperative hemodynamic monitoring. A rise in troponin in a patient at risk for ischemic heart disease has been shown to predict adverse cardiovascular events, designated as a myocardial injury (MINS). An isolated troponin rise warrants attention and should lead to close postoperative hemodynamic monitoring and the implementation of strategies for secondary prevention of myocardial injury. In order to effectively prevent myocardial injury, clinicians need to aggressively control the sympathetically mediated stress response to surgery.
